# Animal Models of Parkinson's Disease

**DOI:** 10.4061/2011/364328

**Published:** 2011-12-18

**Authors:** Yuzuru Imai, Katerina Venderova, David S. Park, Huaibin Cai, Enrico Schmidt

**Affiliations:** ^1^Department of Neuroscience for Neurodegenerative Disorders, Juntendo University Graduate School of Medicine, Tokyo 113-8421, Japan; ^2^Department of Physiology and Pharmacology, Thomas J. Long School of Pharmacy and Health Sciences, University of the Pacific, Stockton, CA 95211, USA; ^3^Ottawa Health Research Institute, Neuroscience Research Institute, 451 Smyth Road, Ottawa, ON, Canada K1H 8M5; ^4^Laboratory of Neurogenetics, National Institute on Aging, National Institutes of Health, 35 Convent Drive, Bethesda, MD 20892-3707, USA; ^5^Department of Bioinformatics and Molecular Genetics and Center for Biological Systems Analysis, University of Freiburg, 79104 Freiburg, Germany

Parkinson's disease (PD) is considered a multifactorial disorder, which is neuropathologically characterized by age-dependent neurodegeneration of dopaminergic neurons in the midbrain. Different neurotoxins including synthetic compounds, heavy metals, and dopamine itself have been proposed to be environmental risk factors of PD. Recent genome-wide genetic and mutational studies provide information on various genetic risk factors while microglial activation in the affected regions has emerged to be involved in the disease development as a local microenvironmental factor. A wide variety of animal models of PD substantially contribute to the understanding of these issues and the development of therapeutic approaches as an alternative to humans although none of them fully recaptures the symptoms and pathology of PD. This special issue is composed of 9 excellent reviews and 3 distinguished original articles that summarize the most recent progresses and ideas obtained from animal models in the pertinent field, while reporting the putative molecular mechanisms of neurodegeneration, therapeutic challenges and limitations using PD models, and generation of new versions of PD models.

The first review paper briefly outlines animal models of PD, covering toxin-induced and genetic models of vertebrate and invertebrate animals, in which characteristic features of each model are discussed.

Mishandling of monoamines including dopamine has been hypothesized to damage neurons. The second review paper describes mice with impaired functions of the vesicular monoamine transporter VMAT2, in which progressive loss of catecholamine-secreting neurons is observed. Such models may be potentially useful for the development of new therapeutic strategies, which would complement current dopamine replacement.

Neuropathological analysis of the postmortem PD brain tissues suggests that an adverse interaction with surrounding glia and other nonneuronal cells may be one of critical steps in neurodegeneration. The third review highlights endotoxin-induced inflammation models, in which activation of microglia and lymphocyte by a bacterial lipopolysaccharide deteriorates a healthy relationship with neurons.

Mutations in the *leucine-rich repeat kinase 2* (*LRRK2*) gene have been identified to cause autosomal-dominant late-onset PD and are also implicated in sporadic PD. The neuropathological features of PD brain tissues with the *LRRK2* mutations are characterized by typical Lewy body pathology in the brainstem. The forth paper reviews a variety of *LRRK2*-related models.

Mutations and increased expression in the **α*-synuclein* gene cause the development of early-onset familial PD. The formation of **α*-synuclein* fibrils and aggregates, a main component of Lewy bodies and Lewy neurites, is considered a key process in the pathogenesis of PD and other synucleinophathies. Other genetic determinants include the genes for Mendelian forms of PD and susceptible genes. The following two papers focus on the potential of *Drosophila* genetic models to examine **α*-synuclein* and other responsible genes. 

Deep brain stimulation (DBS) by electrical pulses could be one of useful therapeutic avenues for PD. However, DBS's technique requires advancement and poor understanding of the mechanisms involved hinder application in clinical practice. The seventh review paper discusses the optimization of a rat PD model for DBS.

Hydrogen has turned out to reduce oxidative damage. The eighth paper introduces the neuroprotective effects of hydrogen on experimental animal models for PD and possible application in treatment and prevention of PD.

The last review explains the limitations of animal models, showing differences between humans and animals, and difficulties in interpretation of obtained results with animal models.

The first research paper investigates selective degeneration of dopaminergic neurons in the substantia nigra and associated motor dysfunction induced by inhalation of mixed manganese compounds on mice. This model could be instrumental for evaluating some aspects of a progressive loss of dopaminergic neurons. The second research paper examines the possible effects of testosterone on PD using a mouse model induced by 1-methy-4-phenyl-1,2,3,6-tetrahydropyridine (MPTP) administration. The study suggests that loss of testosterone induces remodeling in the morphology of medium spiny neurons where dopaminergic neurons of the substantia nigra project although no interaction between testosterone and loss of dopaminergic neurons by MPTP administration is observed. The third research paper of this special issue addresses improvement of potential gene therapy to compensate for impaired complex I activity of the mitochondria using the yeast single-subunit NADH-ubiquinone oxidoreductase, NDI1. NDI1 is functionally able to replace complex I, activity of which is thought to be compromised in most of PD cases.

A decreased sense of smell is one of early signs of PD. Although degeneration of tyrosine hydroxylase-positive neurons in the olfactory bulbs is observed, the pathogenic mechanism underlying olfactory deficits is not well understood. The forth research paper addresses this issue using a rat model bearing the pathogenic **α**-synuclein.

*Yuzuru Imai* *Yuzuru Imai* *Katerina Venderova* *Katerina Venderova* *David S. Park* *David S. Park* *Huaibin Cai* *Huaibin Cai* *Enrico Schmidt* *Enrico Schmidt*

